# Effects of Stores’ Environmental Components on Chinese Consumers’ Emotions and Intentions to Purchase Luxury Brands: Integrating Partial Least Squares-Structural Equation Modeling and Fuzzy-Set Qualitative Comparative Analysis Approaches

**DOI:** 10.3389/fpsyg.2022.840413

**Published:** 2022-04-08

**Authors:** Shaohua Yang, Salmi Mohd Isa, Hongyan Wu, Ramayah Thurasamy, Xi Fang, Yedan Fan, Danping Liu

**Affiliations:** ^1^Graduate School of Business, Universiti Sains Malaysia, Penang, Malaysia; ^2^Department of Business Administration, Daffodil International University (DIU), Dhaka, Bangladesh; ^3^College of Humanities and Management, Guilin Medical University, Guilin, China; ^4^School of Management, Universiti Sains Malaysia (USM), George Town, Malaysia; ^5^Faculty of Economics and Business, Universiti Malaysia Sarawak (UNIMAS), Kota Samarahan, Malaysia; ^6^Fakulti Ekonomi dan Pengurusan (FEP), Universiti Kebangsaan Malaysia (UKM), Bangi, Malaysia; ^7^Department of Management, Sunway University Business School (SUBS), Petaling Jaya, Malaysia; ^8^Faculty of Accounting and Management, Universiti Tunku Abdul Rahman (UTAR), Petaling Jaya, Malaysia; ^9^School of Tourism and Urban-rural Planning, Zhejiang Gongshang University, Hangzhou, China; ^10^School of Management, Xihua University, Chengdu, China

**Keywords:** luxury brand, stores’ environmental components, consumers’ emotions, fsQCA, Chinese consumers, PLS-SEM (partial least squares-structural equation model), purchase behavior

## Abstract

**Purpose:**

Drawing upon the stimulus-organism-response (S-O-R) model, this paper aims to investigate the effects of stores’ environmental components (i.e., lighting, cleanliness, scent, design, layout, music, and employee interaction) on Chinese consumers’ emotions and intentions to purchase luxury brands.

**Design/Methodology/Approach:**

Data were collected from Chinese consumers who have purchased luxury brands from retail stores. Partial least squares-structural equation modeling (PLS-SEM) and fuzzy-set qualitative comparative analysis (fsQCA) were employed for data analysis.

**Findings:**

The results of PLS-SEM indicated that three dimensions of the store environment (i.e., music, scent, and employee interaction) directly and significantly influenced Chinese consumers’ emotions. However, fsQCA revealed greater heterogeneity among respondents by highlighting stores’ environmental components and Chinese consumers’ emotions.

**Originality/Value:**

This study makes significant contributions to the marketing literature by examining the S-O-R model alongside fsQCA approach to conditionally predict outcomes in a luxury brand context. The present study may be one of the first to examine the effects of stores’ environmental components on Chinese consumers’ emotions and purchase intentions, applying both symmetrical test (PLS-SEM) and asymmetrical test (fsQCA) approaches to determine significant components of the store environment as well as component combinations that predict Chinese consumers’ emotions and purchase intentions.

## Introduction

The definition of luxury continues to develop as consumers’ tastes and preferences change. According to Maslow’s hierarchy of needs, individuals’ demand for luxury products spans from a sense of esteem to the need for self-actualization (Shahid and Paul, 2021). Luxury brand consumption has become a crucial part of consumers’ lifestyles and remains an emerging phenomenon among luxury purchase behavior. Luxury consumption can be viewed as a multidimensional concept, composed of financial (i.e., exclusivity), functional (i.e., product excellence), individual (i.e., personal enjoyment), and social value (i.e., prestige and status) (Miller and Mills, 2012; Hennigs et al., 2013). Consumers’ perceptions of luxury brands depend on subjective needs. For instance, consumers are often motivated to purchase luxury brands to illustrate their wealth, social status, and uniqueness (Zhan and He, 2012; Chan et al., 2015; Gentina et al., 2016; Shao et al., 2019). As such, luxury brand practitioners need to understand the numerous psychological motivations that drive consumers to purchase luxury products in their stores.

Given this complex purchase behavior, luxury retailers must be able to not only deliver a product but also build compelling atmospheres (e.g., through the store’s physical environment). Scholars have found that the retail atmosphere exerts noteworthy cognitive, attitudinal, and emotional effects on consumers’ purchase behavior by drawing consumers in, maintaining their engagement and enhancing their purchase experience at a store (Levy and Weitz, 2001; Das, 2014). A store’s atmosphere is built upon sensory elements such as layout, music, and scent; each attribute makes its own contribution to purchase behavior (Chang et al., 2011; Hussain and Ali, 2015; Nusairat, 2015; Raggiotto et al., 2021). These characteristics can be explained using Mehrabian and Russell’s stimulus-organism-response (S-O-R) model, which is grounded in environmental psychology (Nusairat, 2015). The S-O-R model elucidates people’s underlying motivations to purchase or consume luxury brands in terms of stimuli (i.e., environmental factors), the organism (i.e., one’s emotional state), and behavioral responses (i.e., intentions).

The S-O-R model has often been applied in luxury retail contexts to investigate consumers’ purchase behavior. Several studies have outlined the antecedents of purchase intention as well. For instance, a considerable number of academics have investigated the retail store environment as a root cause of purchase behavior along with environmental factors and emotional reactions (Nusairat, 2015; Cho and Lee, 2017). Apart from the store environment, the nexus of consumers’ emotions and purchase intentions is similarly well documented. Key antecedents of purchase intention include a store’s lighting, cleanliness, scent, design, layout, music, and employee interaction (Kim and Moon, 2009; Chang et al., 2011, 2015; Lin and Liang, 2011; Morrison et al., 2011; Mohan et al., 2013; Quartier et al., 2014; Hashmi et al., 2020). Yet such work has primarily aimed to predict emotional outcomes via multiple regression and structural equation modeling; the antecedent conditions for consumers’ emotions remain largely ignored. Consumers’ emotions are inherently complicated and can be influenced by myriad factors. Complexity theory suggests that different causal factors typically play combined roles in affecting an outcome, such that a single causal factor can have either a positive or negative effect. Asymmetrical testing is reflective of this theory and is well suited to explaining consumers’ cognition–affection–conation path toward luxury brand stores.

To fill the above-mentioned knowledge gaps, this study examines the effects of stores’ environmental components (i.e., lighting, cleanliness, scent, design, layout, music, and employee interaction) on consumers’ emotions and intentions to purchase from luxury stores. Second, we apply fuzzy-set qualitative comparative analysis (fsQCA) to examine how to combine elements of the store environment to conditionally predict consumers’ emotions. To fulfill these aims, we employ partial least squares-structural equation modeling (PLS-SEM) to evaluate a measurement model and structural model for theory confirmation. Additionally, we employ fsQCA to discern sufficient and necessary conditions under which Chinese consumers’ emotions are generated. Findings contribute to the luxury brand literature by referring to complexity theory and a combined PLS-SEM/fsQCA approach to offer fresh insight into which configurations of stores’ environmental components can explain consumers’ emotions.

## Theoretical Framework and Hypothesis Development

### Stimuli-Organism-Response Model

The S-O-R model is critical for understanding the nexus between a store’s environmental elements, individuals’ emotions, and behavioral outcomes. This model was derived from the stimuli-response model, which assumes that an expected response will follow from exposure to a predetermined stimulus (Thorndike, 1898). Scholars have argued that humans are dynamic; organismic variables should thus be highlighted between stimuli and responses (Berlo and Gulley, 1957; Woodworth, 1958). The S-O-R model later expanded within environmental psychology. The external environment was identified as an important source of input that elicits individuals’ behavioral responses as output via an organism’s reactions (i.e., emotional states) (Mehrabian and Russell, 1974; Donovan et al., 1994). According to the S-O-R schema in environmental psychology, environmental stimuli extend from the physical environment (e.g., ambience, layout) to the social environment (employees’ emotions and interaction) and emotional states (e.g., pleasure or arousal) that evoke approach or avoidance responses (Bitner, 1992; Donovan et al., 1994; Lin and Liang, 2011). The S-O-R model can also predict consumer behavior under specific circumstances, such as forecasting impulse purchases by identifying the impacts of store-related environmental perceptions based on positive or negative affect (Kim and Moon, 2009; Mohan et al., 2013). The S-O-R model, which covers the dynamics of people’s emotions, responses, and various external environmental cues, serves as the theoretical foundation for the present study. We propose that environmental stimuli (e.g., lighting, cleanliness, scent, design, layout, music, and employee interaction) could influence Chinese consumers’ emotions and spur their intentions to purchase from luxury brand stores.

### Environmental Stimuli and Emotional States

Psychologists have paid extensive attention to environment–behavior relationships. During service encounters, customers are simultaneously exposed to the physical environment and social environment, both of which shape shopping behavior through emotional reactions (Donovan and Rossiter, 1982; Bitner, 1992; Chang et al., 2011). The physical environment consists of ambient elements such as lighting, cleanliness, scent, and music (Baker et al., 1994, 2002; Chang et al., 2011) as well as spatial factors such as design and layout (Bitner, 1992; Tai and Fung, 1997). Environmental cues, including physical items, serve as signals which can convey place-related information to customers explicitly and implicitly (Kim and Moon, 2009). In general, consumers recognize these multiple physical environmental stimuli through sensory feelings and sight, smell, hearing, and touch.

Lighting refers to the arrangement of levels and types of lighting to enhance and illuminate a store’s surroundings and objects (Hashmi et al., 2020). Lighting is one of the most salient environmental stimuli; lighting-level preferences can influence people’s emotional responses (Kurtich and Eakin, 1993; Ryu and Jang, 2007; Quartier et al., 2014). For example, Kurtich and Eakin (1993) found that the type of lighting could affect one’s perceptions of the quality of space physically, emotionally, and psychologically. Moreover, Quartier et al. (2014) provided evidence suggesting that lighting settings can influence the overall atmosphere and emotions, while lighting itself is a signal that communicates a certain image to customers. In luxury brand stores, otherwise called an “M(Art) World,” lighting is adeptly used to deliver messages related to “elegance” or “art” with other aesthetic designs (Joy et al., 2014). Lighting is also an important environmental cue that enhances a store’s “visual quality and comfort” and potentially shapes luxury brand customers’ emotional states (Joy et al., 2014; Hashmi et al., 2020). In light of the previous work, we posit that:

H1: Lighting has a positive effect on Chinese consumers’ emotions in luxury brand stores.

Music is an imperative atmospheric variable; it is directly associated with the in-store experience and customers’ emotional states (Baker et al., 2002; Beverland et al., 2006). Scholars have identified that music in an appropriate genre and playing at a reasonable volume serves as an environmental stimulus that influences consumers’ impressions of brands, evokes emotions, and establishes an emotional bond with customers to ultimately spark behavioral responses (Baker et al., 2002; Grewal et al., 2003; Beverland et al., 2006). Music in luxury brand stores is a critical part of physical ambience, intended to elicit customers’ emotional and behavioral reactions as business rewards (Beverland et al., 2006; Mohan et al., 2013). On this basis, the following hypotheses are proposed:

H2: Music has a positive effect on Chinese consumers’ emotions in luxury brand stores.

Scent is related to “good smells” or “fragrance” and can inspire customer responses such as brand image perceptions, emotions, and behavior (Madzharov et al., 2015; Hashmi et al., 2020; Errajaa et al., 2021). Ambient scent is an integral part of the atmosphere along with music and lighting (Bitner, 1992; Spies et al., 1997; Hashmi et al., 2020). In luxury brand stores, scent is frequently staged as an olfactory marketing strategy to improve customers’ emotional states and strengthen business performance (Madzharov et al., 2015; Errajaa et al., 2021). Madzharov et al. (2015) found ambient scent to be linked with consumers’ preferences and purchase behavior; specifically, in a warm-scented environment, customers displayed stronger brand preferences and more purchases compared with a cool-scented environment. Furthermore, ambient scent that aligns with a brand’s image may positively affect customers’ emotions, satisfaction, and behavioral responses such as purchase intention (Errajaa et al., 2021). Hence, we posit that:

H3: Scent has a positive effect on Chinese consumers’ emotions in luxury brand stores.

Cleanliness is also vital to the overall ambience of luxury brand stores. Hygiene is a core aspect of evaluations of the physical environment, which may influence consumers’ attitudes and behavior toward a brand in general (Joy et al., 2014; Hussain and Ali, 2015). Researchers have documented a relationship between cleanliness and servicescape quality, satisfaction, and emotional states (Fonvielle, 1997; Lucas, 2003). Lucas (2003) pointed out that cleanliness affects customers’ servicescape satisfaction and subsequent behavioral intentions. Relatedly, Hashmi et al. (2020) identified cleanliness as a key component of the store environment, such that environmental cues significantly influence consumers’ enjoyment and emotional states during a shopping experience. Therefore, we put forward:

H4: Cleanliness has a positive effect on Chinese consumers’ emotions in luxury brand stores.

Design is another tangible physical attribute in luxury brand stores. The visual effects of design directly inform customers’ shopping experiences (Spies et al., 1997; Hashmi et al., 2020). Design is particularly related to architecture and decoration, including colors, equipment, and furniture (Hashmi et al., 2020). Superior design caters to a brand’s image to unconsciously influence customers’ brand perceptions (Jiang and Nagasawa, 2014). As mentioned, luxury brand stores have been described as a “M(Art) World” thanks to their aesthetic design. Design essentially includes physical environmental stimuli that exert unconscious but significant impacts on customers’ emotional states and guide behavioral reactions (Baker et al., 2002; Joy et al., 2014; Johnson et al., 2015). The following hypothesis is therefore formulated:

H5: Design has a positive effect on Chinese consumers’ emotions in luxury brand stores.

Layout refers to the arrangement of equipment, furniture, and machinery as well as their spatial relationships, including the sizes and shapes of physical items and objects (Hussain and Ali, 2015; Hashmi et al., 2020). Layout and product locations can influence customers’ movement and shopping pathways immensely. A suitable layout also facilitates product- and information- seeking (Spies et al., 1997; Ryu and Jang, 2007). Luxury brand stores can be deliberately staged to promote the fulfillment of customers’ hedonic needs as well as functional needs. An effective layout shapes customers’ perceptions of the physical environment and consequently influences their mood, emotions, and purchase patterns as behavioral responses (Bitner, 1992; Mohan et al., 2013). Hence, in line with the extensive literature, we hypothesize the following:

H6: Layout has a positive effect on Chinese consumers’ emotions in luxury brand store.

Despite the significant role of the physical environment in consumers’ emotional states and purchase behavior, the social environment – specifically the “human factor” in luxury brand stores – cannot be ignored given its associations with service quality and brand perceptions (Sherman et al., 1997; Baker et al., 2002). Luxury brand stores serve as social places for sales personnel and customers; their interaction embodies social cues and is vital to enhancing service quality and the overall service experience (Hu and Jasper, 2006; Kim and Kim, 2014). Friendly interactions between customers and salespeople, and between customers themselves, also offer psychological and emotional benefits conducive to customer satisfaction and loyalty (Kim and Kim, 2014; Chang et al., 2015). For example, if customers perceive strong arousal or pleasure from social cues (e.g., interaction) in a store environment, they may develop a more favorable brand image (Hu and Jasper, 2006). Hence, interaction embedded in a service encounter is expected to improve a customer’s positive emotional state and to affect their purchase behavior (Kim and Kim, 2014; Hashmi et al., 2020). Based on this logic, we hypothesize the following:

H7: Employee interaction has a positive effect on Chinese consumers’ emotions in luxury brand stores.

### Emotion and Purchase Intention

The body of literature on consumer behavior continues to grow; considerable work in marketing has examined aspects such as customers’ value co-creation behavior and purchase behavior (Graessley et al., 2019; Meilhan, 2019; Watson and Popescu, 2021). Scholars have also sought to identify different features of purchase behavior, including consumers’ purchase-related habits and decisions (Drugãu-Constantin, 2019; Miricã (Dumitrescu), 2019; Rydell and Kucera, 2021). However, to explain purchase behavior more precisely, purchase intention must be determined before consumers engage in specific actions (Ajzen, 1985). Purchase intention is defined as “a situation where [a] consumer tends to buy a certain product in certain condition[s]” (Mirabi et al., 2015) and is considered a key indicator of actual purchases (Ajzen, 1985; Chang and Wildt, 1994).

In the original S-O-R model, one’s emotional state can be described on the basis of three independent dimensions – pleasure, arousal, and dominance – which collectively convey a person’s state of feeling (Mehrabian and Russell, 1974). Pleasure is thought to occupy a continuum ranging from extreme unpleasantness to extreme pleasantness and covering affective feelings such as happiness and satisfaction (Mehrabian and Russell, 1974; Hashmi et al., 2020). Arousal is believed to span from sleep to excitement, reflecting a person’s situational mental state (e.g., when stimulated, excited, or sleepy) (Mehrabian and Russell, 1974; Bakker et al., 2014). One’s emotional state is viewed as an organismic variable representing the impacts of environmental stimuli on behavioral responses; pleasure and arousal are emotional responses and appear closely connected with environmental stimuli and consumers’ purchase behavior (Kim and Moon, 2009; Chang et al., 2011; Morrison et al., 2011; Hashmi et al., 2020).

Abundant research has confirmed a link between emotional state and purchase intention. For example, Donovan and Rossiter (1982) suggested that customers experiencing pleasure derived from the environment may be more likely to return to a store. Ryu and Jang (2007) observed a significant effect of environmental perception on behavioral intention through pleasure and arousal; that is, one’s emotional state influenced their behavioral intention. Beverland et al. (2006) similarly found that given environmental stimulation, an accompanying emotive process affected purchase intention. Morrison et al. (2011) discovered that environmental stimuli could induce pleasure and arousal and thus contribute to approach behavior and a satisfying shopping experience. Hashmi et al. (2020) also found that an emotional state created through a store’s environment could inspire impulse purchases. One’s emotional state is therefore imperative to purchase behavior and purchase intention (Lin and Liang, 2011; Mohan et al., 2013; Hashmi et al., 2020). The following hypothesis is proposed based on the S-O-R model and these findings:

H8: Chinese consumers’ emotions have positive effects on their intentions to purchase from luxury brand stores.

**FIGURE 1:**
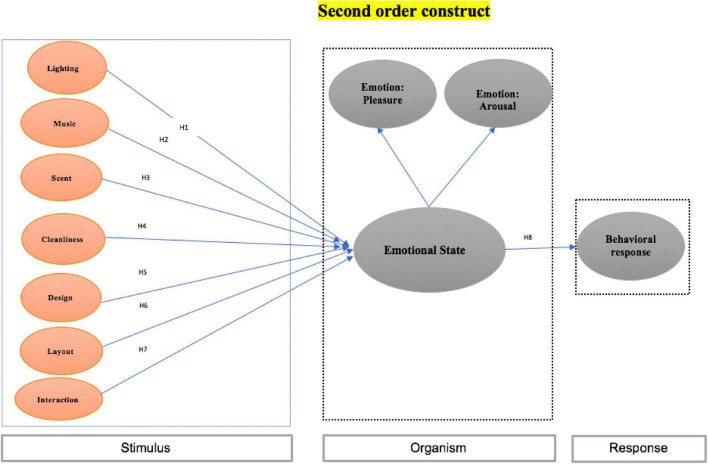
Theoretical framework.

Based on the theoretical and empirical background, the theoretical framework illustrated in Figure 1 depicts the relationships in our study.

## Research Methodology

### Research Site

Luxury brand consumption is a crucial component of modern lifestyles, both in developed nations in the West and emerging markets in the East (Bian and Forsythe, 2012). Most studies on luxury brands have been conducted in developed nations. However, a growing body of work has addressed increasing luxury brand consumption in China (Zhan and He, 2012; Zhang and Kim, 2013). Substantial growth in the Chinese economy and Chinese consumers’ purchasing power have spurred demand for luxury brand consumption (Jin et al., 2021). Western countries are no longer the primary forces behind international luxury brand consumption; China represents the largest potential luxury market (Zhang et al., 2020). The world’s most populous nation has thus become a top candidate for global luxury brand consumption (Kim et al., 2020).

This study focuses on the Chinese context for several reasons. China is home to roughly 1.4 billion people. Numerous international brands have sought to break into this market; luxury consumption is expected to account for roughly 40% of international market share in 2025 (Zhan and He, 2012; McKinsey and Company, 2019). In terms of Chinese consumers’ characteristics, the affluent Chinese are eager to purchase luxury brands to emphasize their social status and prestige (Wang et al., 2011). Luxury brands therefore occupy a critical space in Chinese daily life. Yet to respond to dynamic purchase behavior and thrive in China’s luxury market, the luxury industry must clearly understand Chinese consumers’ behavior. Additionally, China boasts a unique culture and storied history, with a different language and value system than Western countries (Kim et al., 2020). Chinese consumers’ mindset thus varies from that of other consumers. Scholars have provided little insight into marketing strategies for luxury brands in China despite the market’s complexity for international luxury brands (Kim et al., 2020). Against this backdrop, this study seeks to address the managerial and practical utility of Chinese consumers’ luxury retail purchase behavior. Findings are expected to present a holistic picture of Chinese consumers’ purchase behavior and to offer meaningful implications for the luxury brand industry to gain a competitive edge.

### Research Design and Data Collection

A descriptive survey-based research design was adopted to measure all constructs of interest. Data were obtained between June and September 2021 using a quantitative method, namely through a self-administered questionnaire linked via the online platform Wenjuanxing. Potential respondents were identified through non-probability purposive sampling. All respondents were required to have purchased from a luxury brand retail store in the past. Two hundred twenty questionnaires were collected, of which 207 were complete. According to Hair et al. (2006), a sample size ranging between at least 150 and 400 respondents should be sufficient for structural equation modeling. Demographically, 69.6% of respondents were male. Most respondents (73.9%) were between 16 and 25 years old. Only 2.9% of respondents were unemployed; 23.2% worked in the private sector while 20.3% worked in the public sector. Roughly one-third of respondents (34.8%) earned between 10001 and 20000 RMB (1USD = about 6.37 RMB) per month; 20.8% earned more than 40000 RMB monthly. The average income in China is approximately 342,202 RMB per year (Average Salary Survey, 2022).

### Research Instruments

A survey was developed based on prior literature (see Appendix). Five items each measuring lighting and cleanliness were drawn from Han et al. (2011). Three items related to scent were adapted from Hussain and Ali (2015). Design was evaluated based on three items from Baker et al. (2002). Three items were used to assess store layout (Dickson and Albaum, 1977; Mohan et al., 2013). Three items from Baker et al. (2002) constituted the music measurement. Five items were used to evaluate employee interaction (Singh, 2006; Kumar and Kim, 2014). Regarding emotions, six items each measuring pleasure and arousal, respectively, were adapted from Mehrabian and Russell (1974). The purchase intention scale was adapted from Han and Kim (2020). All respondents rated each concept on a seven-point Likert scale anchored by 1 (*strongly disagree*) and 7 (*strongly agree*).

### Statistical Analysis

Data analysis was performed using SPSS, SmartPLS 3.0, and fsQCA. SPSS was employed for data coding and frequency analysis; PLS-SEM (symmetric test) and fsQCA (asymmetric test) were carried out to obtain a richer understanding of the effects of focal components of the store environment on Chinese consumers’ emotions and purchase intentions. PLS-SEM was adopted because our model was prediction-oriented and complex (Hair et al., 2022). The SmartPLS 3.0 software package (Ringle et al., 2015) was used to evaluate our measurement model and structural model (Hair et al., 2019). Subsequently, fsQCA was performed as a complement to PLS-SEM to identify richer outcomes and determine sufficient configurations and causal combinations (Olya and Gavilyan, 2017) of components of the store environment to discern Chinese consumers’ emotions and purchase intentions. Sufficient causal combinations (i.e., configurations) of antecedents were identified in fsQCA 3.0 software to generalize our results (Rasoolimanesh et al., 2021a,b). To ensure sufficient configurations, the consistency and coverage must be greater than 0.8 and 0.2, respectively (Rasoolimanesh et al., 2020).

## Results

### Measurement Model and Structural Model Assessment via Partial Least Squares-Structural Equation Modeling

Following guidelines from Hair et al. (2019, 2022), we evaluated the measurement model by checking the factor loadings (>0.5), composite reliability (CR) (>0.7), and average variance extracted (AVE) to establish reliability and convergent validity. Table 1 lists each construct in the measurement model; all showed adequate reliability and convergent validity. However, the construct of emotion represented a second-order reflective construct. Based on Ringle et al. (2012) recommendations for second-order constructs, emotion was conceptualized in this study as a reflective–reflective second-order construct (i.e., first- and second-order relationships were considered reflective). Twelve items representing two interrelated first-order constructs (i.e., emotion with pleasure and emotion with arousal) corresponded to emotion. After applying a two-stage approach to examine the second-order model (Hair et al., 2022), we found that the factor loadings and CR and AVE values for emotion exceeded the suggested thresholds of 0.5, 0.7, and 0.5, respectively (see Table 1). Therefore, emotion was deemed a second-order construct based on its reliability and validity. Table 2 lists the discriminant validity outcomes using the conservative heterotrait-monotrait (HTMT) ratio approach (Henseler et al., 2015). This ratio should be less than 0.9 to establish discriminant validity (Hair et al., 2022). The measurement model met this criterion.

**TABLE 1:** Measurement model.

1st-order construct	2nd-order construct	Items	Loading	Alpa	CR	AVE
Lighting		Lighting 1	0.853	0.879	0.913	0.681
		Lighting 2	0.907			
		Lighting 3	0.893			
		Lighting 4	0.606			
		Lighting 5	0.832			
Cleanliness		Cleanliness 1	0.952	0.974	0.98	0.906
		Cleanliness 2	0.965			
		Cleanliness 3	0.971			
		Cleanliness 4	0.945			
		Cleanliness 5	0.925			
Scent		Scent 1	0.909	0.912	0.945	0.851
		Scent 2	0.952			
		Scent 3	0.906			
Music		Music 1	0.915	0.828	0.921	0.853
		Music 3	0.932			
Design		Design 1	0.884	0.883	0.927	0.81
		Design 2	0.9			
		Design 3	0.915			
Layout		Layout 1	0.915	0.864	0.917	0.786
		Layout 2	0.861			
		Layout 3	0.883			
Employee		Employee 1	0.706	0.89	0.92	0.699
		Employee 2	0.798			
		Employee 3	0.843			
		Employee 4	0.916			
		Employee 5	0.901			
Pleasure		Pleasure 1	0.777	0.918	0.935	0.706
		Pleasure 2	0.901			
		Pleasure 3	0.897			
		Pleasure 4	0.83			
		Pleasure 5	0.818			
		Pleasure 6	0.81			
Arousal		Arousal 1	0.772	0.872	0.903	0.61
		Arousal 2	0.817			
		Arousal 3	0.852			
		Arousal 4	0.768			
		Arousal 5	0.679			
		Arousal 6	0.789			
	Emotions	Pleasure	0.902	0.818	0.916	0.845
		Arousal	0.936			
Purchase intention		PI1	0.809	0.88	0.917	0.736
		PI2	0.857			
		PI3	0.882			
		PI4	0.881			

**TABLE 2:** HTMT (0.85 or 0.9).

	Cleanliness	Design	Emotion	Employee	Layout	Lighting	Music	Purchase intention	Scent
Cleanliness									
Design	0.716								
Emotion	0.325	0.404							
Employee	0.444	0.634	0.484						
Layout	0.596	0.863	0.435	0.674					
Lighting	0.692	0.666	0.313	0.454	0.575				
Music	0.457	0.683	0.504	0.737	0.721	0.53			
Purchase intention	0.226	0.314	0.513	0.251	0.22	0.313	0.282		
Scent	0.364	0.578	0.424	0.466	0.472	0.516	0.611	0.324	
									

In further accordance with Hair et al. (2022), all hypotheses were tested with 5000 bootstrap samples through a one-tailed test. The PLS-bootstrapping results in Table 3 reveal that lighting (β = −0.034, *p* > 0.05), cleanliness (β = 0.092, *p* > 0.05), design (β = −0.070, *p* > 0.05), and layout (β = 0.103, *p* > 0.05) each had an non-significant positive effect on emotion. Therefore, **H1, H4, H5,** and **H6** were not supported. However, Table 3 also indicates a positive significant effect between music (β = 0.155, *p* < 0.05), scent (β = 0.186, *p* < 0.05), employee interaction (β = 0.194, *p* < 0.05), and emotion. As such, **H2, H3,** and **H7** were supported. Finally, emotion had a highly positive significant influence on purchase intention (β = 0.445, *p* < 0.01), lending support to **H8**.

**TABLE 3:** Path coefficients.

Path model	Beta value	Standard deviation	*t*-value	*p*-value	CILL	CILU
H1 Lighting – >Emotion	−0.034	0.087	0.397	0.346	–0.199	0.092
H2 Music – >Emotion	0.155	0.087	1.781	0.037**	0.012	0.295
H3 Scent – >Emotion	0.186	0.077	2.418	0.008**	0.054	0.309
H4 Cleanliness – >Emotion	0.092	0.111	0.835	0.202	–0.103	0.263
H5 Design – >Emotion	−0.07	0.117	0.597	0.275	–0.28	0.104
H6 Layout – >Emotion	0.103	0.108	0.953	0.17	–0.075	0.277
H7 Employee – >Emotion	0.194	0.078	2.489	0.006**	0.06	0.318
H8 Emotion – >Purchase intention	0.445	0.063	7.006	0.000***	0.328	0.539

***p < 0.001; **p < 0.05; CILL, Confidence Interval Lower Limit; CIUL, Confidence Interval Upper Limit.

Moreover, to verify a model’s overall quality, the value of the coefficient of determination (R^2^) and predictive relevance (Q^2^) should be reported in SEM research (Hair et al., 2019, 2022). We observed that stores’ environmental components (i.e., lighting, music, scent, cleanliness, design, layout, and employee interaction) accounted for 24.6% of the total variance in emotion. In turn, emotion accounted for 19.8% of the variance in purchase intention. Our model therefore possessed satisfactory explanatory power. Exogenous variables also displayed predictive relevance to endogenous variables as reflected by constructs’ Q^2^ values being more than zero (i.e., Q^2^ emotion = 0.169; Q^2^ purchase intention = 0.138).

### Fuzzy-Set Qualitative Comparative Analysis

We adopted fsQCA as a complementary method for two reasons: (1) factors’ inter-variable correlations were all lower than 0.8; and (2) contrasting cases were discerned.

The first step of fsQCA entails the calibration of original construct scores, which should be transformed to set measures (Ragin, 2008). We used the direct method of calibration as suggested by Ragin (2008) to identify three breakpoints (full non-membership, full membership, and a crossover point for maximum membership ambiguity) in each construct’s calibrations. Normally, the original construct scores in the highest quintile are calibrated to 0.9 membership, those in the middle quintile are transformed to 0.5 membership, and those in the lowest quintile are denoted by 0.05 membership.

On the basis of complexity theory (Woodside, 2013, 2014), fsQCA uses set theory to explain causal conditions that may predict a certain outcome. Thus, consistency and coverage are key fsQCA indices. After several tests and adjustments, the configurations that applied to at least one case with a consistency cut-off point of 0.88 in the truth table were submitted for further analysis (Fiss, 2011; Woodside, 2013). After testing the sufficient configurations, we applied a consistency level higher than 0.8 and a coverage level greater than 0.3 to ensure that the combinations of causal conditions were useful (Ragin, 2008; Woodside, 2013; Pappas and Woodside, 2021). The fsQCA output contains three types of solutions (complex, parsimonious, and intermediate) as potential combinations and configurations that can predict strong outcome conditions in most cases. Only parsimonious and intermediate solutions were considered in this study to focus on peripheral and core causal conditions in the results (Ragin, 2008; Fiss, 2011).

Regarding the prediction of strong customer emotion, the fsQCA results in Table 4 point to two useful solutions (i.e., sufficient configurations). In each solution, cleanliness, scent, and store layout constituted core causal conditions. Lighting and design were peripheral in the first solution (Lighting*Cleanliness*Scent*Design*Layout), while design and employee interaction were peripheral in the second solution (Cleanliness*Scent*Design*Layout*Employee). Both solutions were empirically relevant in that they produced high levels of customer emotions; their raw coverage was greater than 0.3, and their overall consistency was greater than 0.851. Music was not a peripheral condition in predicting strong emotion in either solution. Finally, more heterogenous combinations of elements of the store environment could generate Chinese consumers’ emotions compared to the symmetric results of the PLS-SEM method.

**TABLE 4:** Core-periphery conditions producing strong emotions.

Causal configuration/solutions predicting high scores in emotion	Raw coverage	Unique coverage	Consistency
Lighting*Cleanliness*Scent*Design*Layout	0.33	0.20	0.87
Cleanliness*Scent*Design*Layout*Employee	0.32	0.31	0.87
Overall consistency	0.85		
Overall coverage	0.47		

*Causal factors enclosed by borders are core conditions; causal factors without borders are peripheral conditions. The factor of Music was excluded because it was neither a core nor peripheral condition.*

## Discussion

We sought to investigate the effects of stores’ environmental components (i.e., lighting, cleanliness, scent, design, layout, music, and employee interaction) on Chinese consumers’ emotions and intentions to purchase from luxury brand stores. This study also aimed to further examine how combinations of elements in the store environment could predict strong consumer emotions. To achieve these objectives, we explored the influences of environmental components on consumer outcomes (i.e., emotion and purchase intention) via PLS-SEM and fsQCA. Findings partially validated the S-O-R model and illustrated various effects of environmental store components on emotion and purchase intention.

First, the PLS-SEM results demonstrated significant effects of music, scent, and employee interaction on Chinese consumers’ emotions. In line with previous research (i.e., Lin and Liang, 2011; Morrison et al., 2011; Hashmi et al., 2020), these three elements appeared to predict emotion. For example, Chinese consumers could be excited and happy while shopping in luxury brand stores while listening to music, perceiving pleasant smells, and experiencing high-quality customer service. PLS-SEM also indicated that lighting, cleanliness, design, and layout insignificantly predicted Chinese consumers’ emotions. These patterns were inconsistent with earlier literature (i.e., Kim and Moon, 2009; Chang et al., 2011; Quartier et al., 2014; Hashmi et al., 2020), presumably due to different research settings and sample characteristics. In terms of luxury retail settings in China, Chinese consumers can experience pleasure or arousal without being concerned about the in-store environment (e.g., lighting, cleanliness, design, and layout). Furthermore, PLS-SEM indicated that the relationship between Chinese consumers’ emotions and purchase intentions was highly significant. This result is congruent with several studies in contexts including online purchases (Xu et al., 2020), mobile app use (Martinez, 2017), and Pakistani shops (Aziz et al., 2018). Chinese consumers seemed to have strong intentions to purchase luxury products from a retail store when experiencing emotions such as pleasure and arousal. Overall, our PLS-SEM findings partially supported the applicability of the S-O-R model to luxury brand stores in China.

Second, whereas PLS-SEM only unveiled the main (net) influences of antecedents on consequences, fsQCA showed sufficient combinations of antecedents. This complementary approach rendered our findings more comprehensive. To supplement fsQCA, we also identified several heterogeneous combinations (solutions) of possible antecedents related to stores’ environmental components, which could evoke Chinese consumers’ emotions. The fsQCA results suggested that two groups of combination dimensions from the store environment could conditionally predict Chinese consumers’ emotions. We observed five sufficient combinations of the store environment (i.e., lighting, cleanliness, scent, design, and layout) that predicted Chinese consumers’ intentions to purchase from luxury brand stores. We similarly noted five sufficient combinations of the store environment (i.e., cleanliness, scent, design, layout, and employee interaction) that could predict Chinese consumers’ purchase intentions. Interestingly, these findings differed slightly from those of PLS-SEM; hypothesis testing indicated that lighting, cleanliness, design, and layout had no power to predict emotion. The S-O-R model may therefore be challenging to examine using a multifaceted and multimethod approach. Therefore, a symmetric test cannot provide a sufficiently generalized understanding of Chinese consumers’ purchase behavior.

The fsQCA results showed the most important components of the store environment to be cleanliness, scent, and layout, which clearly stirred Chinese consumers’ emotions compared with other components. These findings partially opposed those of PLS-SEM as well. This discrepancy may be attributable to the context-based nature of environmental store components. Finally, the fsQCA results stressed that music is not important to consider, distinct from the PLS-SEM results that suggested its significant effect.

## Conclusion

### Theoretical Contributions

First, this study contributes to the conceptual enrichment of stores’ environmental components. Prior literature only presented a typology of the store environment based on ambient, design-related, and social factors (Lin and Liang, 2011; Kumar and Kim, 2014; Nusairat, 2015), potentially hindering a more holistic understanding of stores’ environmental cues. Our work expands the marketing literature by incorporating numerous elements (i.e., lighting, cleanliness, scent, design, layout, music, and employee interaction) as predictors in a luxury brand context to provide a fresh perspective on associations among the store environment and Chinese consumers’ emotions. This study also contributes to the marketing literature by confirming the suitability of the S-O-R model (Mehrabian and Russell, 1974) in a luxury brand context: we conceptualized each category as either a stimulus (i.e., lighting, cleanliness, scent, design, layout, music, and employee interaction), organism (emotion), or response (behavioral intention) and generalized relevant cause–effect relationships.

Second, integrating PLS-SEM with fsQCA produced novel insight. Our findings only partially validated the S-O-R model but illuminated distinct influences of stores’ environmental components on Chinese consumers’ emotions. Therefore, this study extends the luxury brand literature by drawing upon complexity theory (Woodside, 2013, 2014) and applying a unique asymmetric method (fsQCA) to Chinese consumers’ emotions toward luxury brands. The results of linear regression and fsQCA revealed crucial antecedents of these consumers’ reactions to luxury brands. These approaches also generated some incongruent findings, offering more in-depth insight into stores’ environmental cues and Chinese consumers’ emotions.

### Practical Implications

**FIGURE 2:**
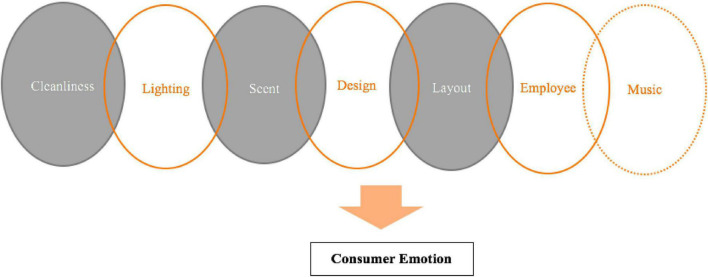
Strong store environmental components predicting consumer emotion (fsQCA result).

This study provides several implications. The findings offer a useful framework for luxury brand stakeholders wishing to improve stores’ environmental elements and to highlight the roles of these elements. Luxury store managers should consider the importance of environmental components and Chinese consumers’ emotions rather than simply concentrating on product- or brand-oriented marketing. Results also suggest that two components, scent and employee interaction, should be given additional attention when store managers seek to craft an appealing environment to enhance consumers’ emotions. Moreover, combinations of lighting, cleanliness, scent, design, and layout or of cleanliness, scent, design, layout, and employee interaction should promote Chinese consumers’ pleasure and arousal while shopping with luxury retailers. Therefore, store managers should strive to craft an appealing environment through enhanced cleanliness, scent, and layout (Table 4 and Figure 2). For example, stores should be clean, have a pleasant aroma, and present items in an orderly fashion. Finally, Chinese consumers’ pleasure and arousal should be prioritized while shopping; our results suggest a positive association between emotion and purchase intention. Stores’ marketing strategies should be developed to make consumers happy and thus boost their intentions to purchase luxury products in-store.

### Limitations and Future Work

This study has certain limitations which offer avenues for future work. Our sample only included 207 respondents, which may not reflect a larger geographical area. Scholars could use larger samples to improve theoretical generalizability in the future. Moreover, luxury brands include Louis Vuitton, Gucci, Prada, and others. Purchase intention–related bias may exist based on product type. Subsequent investigations could employ multigroup analysis to compare Chinese consumers’ intentions to purchase specific luxury brands (e.g., Louis Vuitton vs. Gucci). Further, our research model was rather simplistic and did not consider indirect relationships among constructs given fsQCA’s constraints in exploring conditional indirect effects. Once fsQCA can do so, researchers will be able to identify antecedent conditions of emotions and behavior. Lastly, this study did not analyze demographic variables such as gender and age. Differences in these variables among Chinese consumers could lead to substantial variation in purchase behavior. Therefore, future studies could introduce demographic features (e.g., gender and age) into a measurement model as control variables.

## Data Availability Statement

The original contributions presented in the study are included in the article/supplementary material, further inquiries can be directed to the corresponding author.

## Author Contributions

SY and HW: conceptualization. SY: writing – original draft and proofreading. YF: writing – literature review. SMI and RT: reviewing, revising, and supervision. XF and SY: data collection, research methodology, and data analysis. HW: project administration. HW and DL: editing and formatting. All authors contributed to the article and approved the submitted version.

## Conflict of Interest

The authors declare that the research was conducted in the absence of any commercial or financial relationships that could be construed as a potential conflict of interest. The reviewer MKH declared a shared affiliation with the authors, RT, SY, and SMI, to the handling editor at the time of review.

## Publisher’s Note

All claims expressed in this article are solely those of the authors and do not necessarily represent those of their affiliated organizations, or those of the publisher, the editors and the reviewers. Any product that may be evaluated in this article, or claim that may be made by its manufacturer, is not guaranteed or endorsed by the publisher.

## Appendix

**TABLE A1:** Measurement.

Construct and Measurement Items	No. of Items	Sources
**Lighting**	5	Han et al., 2011
I prefer the light in store that allows me to evaluate the quality of product clearly.		
Sufficient of lighting at the corners of store let me see clearly.		
I am attractive to the store with sufficient lighting.		
Different lighting used in store attracts my attention.		
I feel comfort with bigger clarity in store.		
**Music**	3	Baker et al., 2002
The store had pleasant music.		
The store had bothersome music.		
The store had appropriate music.		
**Scent**	3	Hussain and Ali, 2015
Scent in retail chain outlet encourages me to purchase more.		
Scent in the store makes me to revisit retail chain outlet.		
Fragrance of the retail chain outlets makes me to stay more time.		
**Cleanliness**	5	Han et al., 2011
I prefer store with clean floor.		
I prefer store with clean shelves.		
I prefer store that is always in clean condition.		
The products in store are tidy in arrangement.		
I feel comfort in the clean and tidy store.		
**Design**	3	Baker et al., 2002
The store has pleasing color scheme.		
The store has the attractive facilities.		
The store has well organized merchandise.		
**Layout**	3	Dickson and Albaum, 1977; Mohan et al., 2013
It was easy to move about in the store.		
It was easy to locate products/merchandise in the store.		
The store had attractive displays.		
**Interaction**	5	Singh, 2006; Kumar and Kim, 2014
There were enough employees in the store to service customers.		
The employees were well-dressed and appeared neat.		
The employees were friendly.		
The employees were helpful.		
The employees were knowledgeable.		
**Pleasure**	6	Mehrabian and Russell, 1974
Unhappy–Happy		
Annoyed–Pleased		
Unsatisfied–Satisfied		
Melancholy–Content		
Despairing–Hopeful		
Bored–Relaxed		
**Arousal**	6	Mehrabian and Russell, 1974
Unaroused – Aroused		
Relaxed – Stimulated		
Calm – Excited		
Sleepy – Wide-Awake		
Sluggish – Frenzied		
Dull – Jittery		
**Behavioral response (purchase intention)**	4	Han and Kim, 2020
I will convey positive opinions about my experience with this luxury brand.		
In other situations, I will purchase additional products/services from this luxury brand.		
I will continue to use this luxury brand.		
I am willing to recommend this luxury brand to others.		
